# Hydrodynamic Cavitation as a Method of Removing Surfactants from Real Carwash Wastewater

**DOI:** 10.3390/molecules29204791

**Published:** 2024-10-10

**Authors:** Magdalena Lebiocka, Agnieszka Montusiewicz, Elżbieta Grządka, Sylwia Pasieczna-Patkowska, Jerzy Montusiewicz, Aleksandra Szaja

**Affiliations:** 1 Faculty of Environmental Engineering, Lublin University of Technology, Nadbystrzycka 40 B, 20-618 Lublin, Poland; a.montusiewicz@pollub.pl (A.M.); a.szaja@pollub.pl (A.S.); 2 Faculty of Chemistry, Institute of Chemical Sciences, Maria Curie Skłodowska University, Pl. Marii Curie-Skłodowskiej 3, 20-031 Lublin, Poland; elzbieta.grzadka@mail.umcs.pl (E.G.); sylwia.pasieczna-patkowska@mail.umcs.pl (S.P.-P.); 3 Faculty of Electrical Engineering and Computer Science, Lublin University of Technology, Nadbystrzycka 38A, 20-618 Lublin, Poland; j.montusiewicz@pollub.pl

**Keywords:** car wash wastewater, surfactants, hydrodynamic cavitation, industrial wastewater, multi-criteria decision support

## Abstract

The present work aimed to evaluate whether the use of an innovative method such as hydrodynamic cavitation (HC) is suitable for the simultaneous removal of surfactants of different chemical natures (non-ionic, anionic and cationic) from actual car wash wastewater at different numbers of passes through the cavitation zone and different inlet pressures. An additional novelty was the use of multi-criteria decision support, which enabled the selection of optimal HC conditions that maximized the removal of each group of surfactants and chemical oxygen demand (COD) with minimal energy input. For the optimal HC variants, Fourier transform infrared spectroscopy (FT-IR/ATR) as well as investigations of surface tension, zeta potential, specific conductivity, system viscosity and particle size were carried out. The highest reduction of non-ionic surfactants was found at 5 bar inlet pressure and reached 35.5% after 120 min. The most favourable inlet pressure for the removal of anionic surfactants was 3 bar and the removal efficiency was 77.2% after 120 min, whereas the most favourable inlet pressure for cationic surfactant removal was 3 bar, with the highest removal of 20% after 120 min. The obtained results clearly demonstrate that HC may constitute an effective, fast and cost-efficient method for removing surfactants from real industrial wastewater.

## 1. Introduction

Car washes are one of the most important services in urban areas, with both automatic and manual (self-service) facilities. A large amount of water is used to wash vehicles, generating significant amounts of wastewater, averaging around 150 L per car for the first and 400 L for the second type of facility [1]. Currently, the problem of wastewater from car washes is also exacerbated by the exponentially growing number of car washes and vehicles used by owners [2], as well as by the presence of persistent organic pollutants, which may pose an additional risk to the environment due to the expanded scope of the problem. The composition of the wastewater varies depending on the number of cars washed, the substances used to clean them, the climatic conditions, the region/continent and the type of dirt. In addition to sand, gravel, total suspended solids (TSS) and dispersed particles causing turbidity, the wastewater contains potentially hazardous pollutants, including detergents, degreasers, waxes, brighteners, alkaline/acid cleaners, solvents, hydrocarbon residues and heavy metals from paint residues [3,4]. The values of the quality parameters are generally in a wide range, with the most commonly reported factors including chemical oxygen demand (COD), TSS, total dissolved solids (TDS) and turbidity, as well as oil and grease, with the respective amounts being 67–8190 mg L^−1^, 49–5856 mg L^−1^, 520–17,268 mg L^−1^, 20–3649 NTUs (Nephelometric Turbidity Units) and 6–550 mg L^−1^ [5], with the upper values being characteristic of rubbish lorry washing. Surfactants are surface-active compounds that are frequently used in vehicle washing. High concentrations of these pollutants are present in wastewater after car washing, and they are considered to be the most critical pollutants. Their reported levels are in a wide range of 2–290 mg L^−1^, with the lowest values for car wash wastewater from Africa and North America and the highest for Europe [6]. Surfactants efficiently reduce the surface tension of liquids, which is due to amphiphilic structures, which have both hydrophobic and hydrophilic parts, within their molecules [7,8]. Depending on their charge, they can be categorised into four groups based on their hydrophilic part: non-ionic (0), anionic (−), cationic (+) and amphoteric/zwitterionic (±) [9]. Surfactants are used in many applications as cleaning agents, foaming agents, emulsifiers, wetting agents and dispersing agents [10]. Among them, anionic and non-ionic surfactants are used most frequently, as they are the main components of detergents. Cationic surfactants are the most toxic. Nevertheless, due to their low production (it is estimated that their global production does not exceed 7–10%), their threat to the environment is negligible. Typically, car wash wastewater is released into the sewerage system without undergoing any form of treatment [11,12]. Thus, developing reliable systems capable of improving car wash wastewater treatment to enable its recovery is vital. Many technologies are currently being investigated to fulfil this requirement. These include membrane separation [13,14], advanced electrochemical oxidation processes, e.g., the electro-Fenton process and electrooxidation with hydrogen peroxide generation [15], electrochemical treatment with Fe and Al electrodes [16], and various hybrid systems. Among them, the combination of coagulation–flocculation with adsorption and sedimentation [17], microfiltration with ceramic membranes and reverse osmosis [18], the integration of coagulation–flocculation with sand filtration, the combination of electrocoagulation with nanofiltration [19] or the coupling of microbial fuel cells with metal–organic frameworks [20] are of particular interest. Comprehensive overviews of the subject area have also been presented by Espinoza-Montero et al. [4] and Dadebo et al. [6]. Among the various methods for removing surfactants, hydrodynamic cavitation (HC) appears to be a promising solution. The phenomenon of cavitation takes place when micro vapor bubbles are created within a flowing liquid, ascend and subsequently collapse as a result of an abrupt decrease in local pressure. Cavitation can be classified into four types based on how it is generated: hydrodynamic, acoustic, optical and molecular. To date, only the hydrodynamic and acoustic types of cavitation have been shown to result in physico-chemical changes that are desirable in pretreatment processes [21,22], while the latter is considered more efficient due to its ability to oxidise organic substances combined with low energy and operating costs [23,24]. HC is an effective technique which is of interest in many scientific fields, including water/wastewater treatment and disinfection [25,26,27], water reclamation [28], cell and lignocellulosic fibre disruption [29,30], biodiesel synthesis [31,32], extraction [33] and emulsion production [34], as well as the degradation of resistant organic compounds (including pharmaceutical residues, pesticides, textile dyes and phenols) [35,36,37,38]. The feasibility of using HC to remove surfactants was investigated by Mukherjee et al. [39], focusing on sodium dodecyl sulphate’s (anionic surfactant) degradation in distilled water, and Pereira et al. [40], who looked at wastewater from the manufacture of tattoo ink; both studies used a similar HC device. As far as the authors are aware, the presented manuscript is the pioneering work concerning the removal of surfactants from real car wash wastewater using hydrodynamic cavitation. The tested car wastewater contained high concentrations of surfactants, but their properties differed depending on when they were collected. The novelty of this work is the multi-criteria decision support that introduces a multi-criteria analysis to enable the selection of optimal HC conditions that maximise the removal of each group of surfactants and COD with minimum energy input. To evaluate the suitability of HC for surfactant removal at different inlet pressures and numbers of passes through the cavitation zone, their concentration was analysed. For the optimal HC variants, real car wash wastewater samples were additionally studied by Fourier transform infrared spectroscopy with an attenuated total reflectance (FT-IR/ATR) accessory, and the surface tension, zeta potential, specific conductivity, viscosity and particle size of the measured samples were established as well.

## 2. Results and Discussion

### 2.1. HC for Surfactant Removal from Carwash Wastewater

When analysing the composition of the untreated wastewater, it should be noted that, compared to the data presented by Dadebo et al. [6], these samples show a low COD and low turbidity, a slightly alkaline pH and a relatively high surfactant concentration. Accordingly, surfactant concentration was recognised as a key factor in evaluating the usefulness and efficiency of HC. According to Mukherjee et al. [39], lower degradation rates are obtained at higher pollutant concentrations. This is due to the fact that as the initial concentration increases, the total number of contaminant molecules increases, whereas the concentration of •OH radicals remains constant in the system. The mechanism of reactive radicals is based on their ability to oxidize organic pollutants, either directly or through interactions with dissolved oxygen [41]. These reactions can occur in both homogenous and (micro)heterogeneous environments.

**Figure 1:**
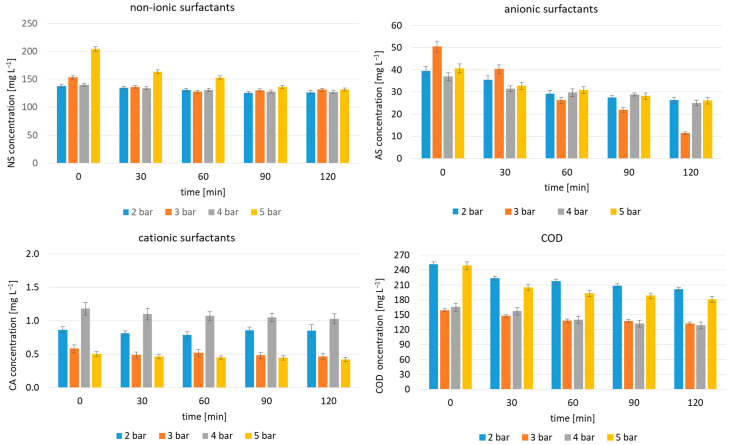
Changes in surfactant concentrations and COD (average values, error bars represent 95% confidence limits for means).

**Figure 2:**
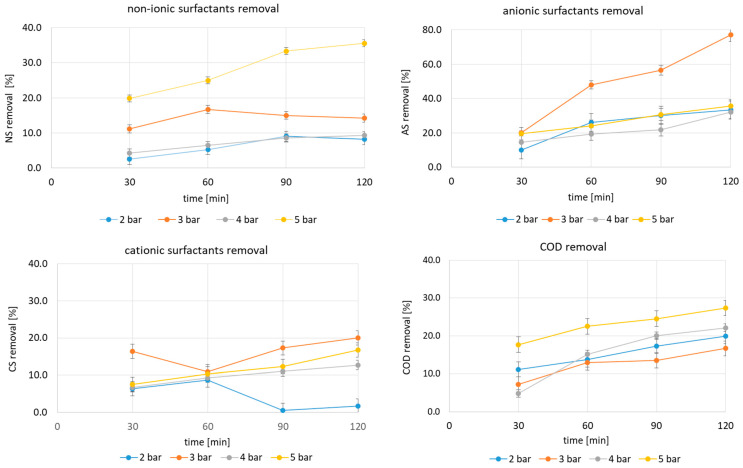
Removal efficiency in surfactant concentrations and COD (average values, error bars represent 95% confidence limits for means).

HC’s efficiency in degrading car wash wastewater at the assumed operating variables, i.e., a fixed inlet pressure and test duration, was studied and the obtained results are presented in Figure 1 and Figure 2. The changes in the concentration of cationic, anionic and non-ionic surfactants and COD are shown in Figure 1. The corresponding removal efficiency is shown in Figure 2.

In all cases, the surfactants were degraded via HC, albeit to varying degrees for the specified group of pollutants, governed by test duration and inlet pressure. The highest reduction of non-ionic surfactants was observed at 5 bar and reached 35.5% after 120 min of the process. When using 3 bar, the highest HC efficiency was observed at 60 min (16.7%), after which it decreased slightly and remained at a comparable level until the experiment terminated. The lowest decreases for the non-ionic surfactants were observed at inlet pressures of 2 and 4 bar. For the removal of anionic surfactants, the most favourable inlet pressure was 3 bar. The removal efficiency increased with HC time and the highest value of 77.2% was reached at 120 min. At 5 bar, the same trend was observed, but the removal efficiency of the anionic surfactants was halved at 120 min compared to 3 bar. For this group of surfactants, inlet pressures of 2 and 4 bar proved to be the least effective. A similar trend was observed for cationic surfactants. The most favourable inlet pressure was 3 bar with the highest removal of 20% after 120 min of processing. In summary, ionic surfactants were degraded more efficiently at a lower inlet pressure of 3 bar (cv = 0.12) than non-ionic surfactants, for which 5 bar was found to be favourable (cv = 0.07). COD removal was also highest with 5 bar, achieving 27%. According to Pereira et al. [40], the maximum efficiency of surfactant degradation (37%) by HC was found at 4.5 bar and an inlet orifice diameter of 1.5 mm, but it involved a significantly lower concentration of the surfactant, amounting to 3.2 mg L^−1^ and acidic conditions (pH = 2.5). In turn, Mukherjee et al. [39], who looked at HC for anionic surfactants at concentrations of 2–15 mg L^−1^, showed the highest removal (64.9%) at 5 bar, at a concentration of 10 mg L^−1^ and pH = 2.0 and with a 1.6 mm diameter inlet hole.

Turbidity increased due to cavitation with inlet pressure and HC duration, which was due to particle size reduction. Not surprisingly, the highest turbidity increase of 54% was observed at 5 bar after 120 min, while the lowest values were at pressures of 2 and 3 bar and did not exceed 7% and 11%, respectively. An analogous trend was observed for the phosphate concentration, with a highest phosphate release of 67.6% (5 bar, 120 min) and lowest of 20.7% (2 bar, 120 min), which was probably related to the degradation of the surfactant. The values observed for both the non-cavitated and cavitated samples were low. The mechanism of surfactant degradation is a direct consequence of the HC process, which creates tiny vapor bubbles by passing the liquid through specific geometries, causing a rapid pressure drop. These bubbles expand and then implode due to the surrounding fluid pressure, leading to a temperature rise and a pressure increase. This collapse pyrolyzes water molecules into ·H and ·OH radicals, which are strong oxidizing agents that degrade surfactants molecules [39].

### 2.2. Multi-Criteria Decision Support of HC Process

Due to the complexity of the experimental data as well as the plethora of decision variables, typical inference methods proved useless in determining which of the runs could be recommended as the best solution. In order to select the optimal HC conditions that led to the best surfactant removal results with the lowest possible energy demand, a multi-criteria analysis was performed for 20 detailed variants (Table 1). Table 2 shows the assumptions made and the obtained results.

Assuming that all criteria, i.e., the concentration of cationic, anionic and non-ionic surfactants as well as the COD and energy consumption are equally important from a technological point of view (stage AS-2/1), variant 10 (v10) was optimal, for which the following operating conditions applied: 3 bar inlet pressure and a circulation time of the wash wastewater in the cavitation system of 120 min. In practice, the individual criteria should be weighted differently due to their respective environmental impact and the cost-effectiveness of the HC method. Despite the assumed different weighting, the same variant was indicated as optimal (v10) by the multi-criteria analysis in all cases. This was shown both when the concentrations of non-ionic and anionic surfactants (w_1_ = 0.2, w_2_ = 0.3) and energy consumption (w_5_ = 0.25) were considered as key factors for the cavitation of car wash wastewater (level AS-2/3) and when energy consumption (w_5_ = 0.6) was considered as the most important criterion (level AS-2/3). In addition, the same variant was selected as optimal when the concentration of non-ionic surfactants (w_1_ = 0.5) was considered critical for process efficiency due to their high concentration in the wash water (stage AS-2/4). The optimum first-order solution was therefore variant 10, which was associated with the lowest increases in turbidity and phosphates of 11% and 30%, respectively. In the seven-criteria analysis, where the above-mentioned variables were included as additional criteria to be minimised, the same variant was found to be optimal (unpublished data).

It was also interesting to find another suboptimal variant beyond v10 by determining the optimal second-order solution (suboptimal solution) and keeping the same weights as previously assumed. This time, variant 17 (v17) was given, which was the same in each analysis of both min-max type (level AS-3/1) and min-max type with weights (levels from AS-3/2 to AS-3/4), although the variable weights of the criteria were used (Table 1). The aim was to carry out the trial at 5 bar for 30 min, which was accompanied by a higher increase in turbidity and phosphates of 43% and 37%, respectively. The specialist’s task is to choose between the v10 and v17 variants, taking into account the preferences. In view of the above results, inlet pressures of 3 and 5 bar were considered for the following detailed analysis of the use of HC to remove surfactants from car wash wastewater.

### 2.3. Physicochemical Analysis of the Wastewater under Optimal HC Conditions

Measuring surface tension is an excellent method for monitoring changes in surfactant concentration in the systems under investigation. As you can see in Table 3 below, the surface tension increases with the duration of the cavitation process, which means that the concentration of surfactants in the analysed systems gradually decreases. This is proof of the effective removal of surfactants during hydrodynamic cavitation. After 120 min, the surface tension in both series of measurements (3 bar; 5 bar) is comparable to that of pure water (72.8 mN/m at 25 °C [42]), which proves that the measured systems are almost free of all surfactants.

The pH value of the measured systems increases with the duration of cavitation. This increase may be due to the presence of more basic groups in the system, the decay of acidic groups or the effect of the gas nanobubbles released during hydrodynamic cavitation [26]. The changes in pH over time are also greater at higher pressure, which is due to the higher concentration of nanobubbles released. Interestingly, the conductivity decreases with the duration of cavitation at both tested pressures, indicating the loss of ionic species in the solution. The fact that the pH increases in each tested series most likely signifies a significant loss of acidic forms in the examined systems. As for the zeta potential, its values decrease with the duration of cavitation in both series. A more negative zeta potential means a greater concentration of negatively charged groups in the systems [43], corroborating the observations made during the measurements of pH. The changes in the zeta potential values are greater in the 3 bar system, which is most likely due to the higher initial concentration of surfactants in this system compared to the 5 bar system.

Analysing the changes in the viscosity of the systems during the cavitation process allows obtaining additional data concerning the qualitative composition of the tested systems. A higher viscosity means that more substances can influence the internal friction caused by the sliding of liquid layers relative to each other during the flow [44]. In the systems tested, slight changes in viscosity were observed in both examined series, but these changes were so small that it can be concluded that the viscosity of the wastewater changes only insignificantly in the course of the cavitation processes.

The particle size (z-ave) of the wastewater samples varies depending on the duration of cavitation. The gas nanobubbles released during this process lead to a significant decrease in particle size, which can be observed in both test series. The longer the cavitation time, the more molecules were torn apart, so that their size also decreased. The use of a higher pressure allows a more effective change in particle size, but it should be borne in mind that the initial samples, as real wastewater, have different compositions, which affects the initial values of the particle sizes in the system.

### 2.4. FT-IR/ATR Analysis under Optimal HC Conditions

**Figure 3:**
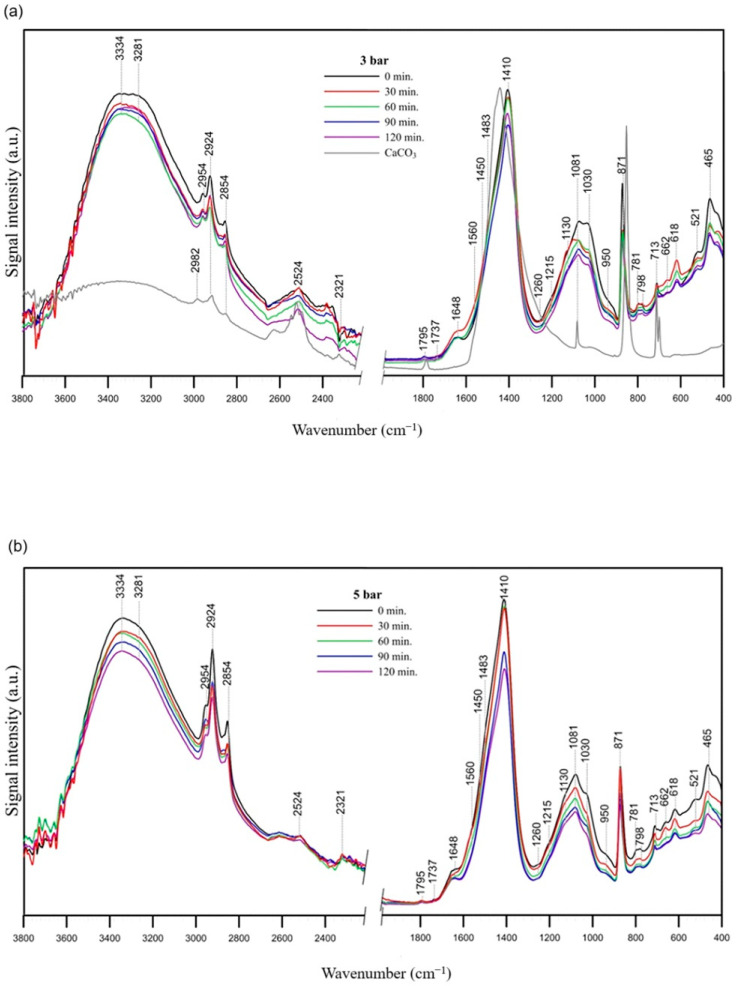
FT-IR/ATR spectra of the studied car wash wastewater samples before (0 min) and after cavitation treatment (30–120 min), subjected to cavitation at two different pressures: (**a**) 3 bar, (**b**) 5 bar.

FT-IR spectroscopy was used to study the impact of hydrodynamic cavitation on the fragmentation and decomposition of substances present in car wash wastewater. Before recording the IR spectra, the wastewater samples were freeze-dried, as the water contained in the samples causes the formation of broad and intense bands in the spectra, which would make their correct interpretation impossible. The spectra of the cavitated and subsequently freeze-dried wastewater of both the initial samples and the samples that were subjected to cavitation at two optimal pressures (3 and 5 bar) are shown in Figure 3.

The composition of solid residues in car wash wastewater is complex and varied and can change over time, depending on the season and the location of the car wash, among other factors. Nevertheless, the presence of surfactants, phosphorus and nitrogen compounds, oils and greases as well as additives (dyes, fragrances, plasticisers, substances to protect against re-soiling, anti-corrosion additives and preservatives) is to be expected [45]. In addition, the suspended matter may also contain calcium carbonate as a component of the water used for washing.

The shape of the spectra and the occurrence of certain bands in the spectra of the initial wastewater (Figure 3a,b) indicate that their composition is similar but not identical. All spectra show bands with two maxima at about 3350 and 3280 cm^−1^, indicating the presence of -OH stretching vibrations in acids and alcohols and/or N-H stretching vibrations. Hydrocarbon chains are visible as strong bands at 2954, 2924 and 2850 cm^−1^ as a result of the C–H stretching of methyl and methylene groups. The bands at ~713 and 1450 cm^−1^ result from CH_2_ rocking vibrations and CH_2_ scissoring deformation, respectively. The former could also indicate the presence of carbonates, as do the peaks at ~870, 1083, ~1410, 2524 cm^−1^. The band at 1410 cm^−1^ is also characteristic of -OH bending in alcohols [46] and/or S=O stretching vibrations of undissociated -SO_3_H groups from anionic surfactants [47]. The presence of the low intensity bands at ~1260, 1215 cm^−1^ and the band at ~1080 cm^−1^ also indicates the symmetric S=O stretching of the head group of an anionic surfactant, probably sodium lauryl sulphate (SLS) or sodium laureth sulphate (SLES). However, the band at 1080 cm^−1^ might contain a contribution from C–O stretching and carbonate vibrations, as mentioned above. The low intensity peak at ~1483 cm^−1^ (shoulder) may be connected to scissoring vibrations of CH_3_-N^+^ moieties [48] in cationic surfactants. The peak at ~1650 cm^−1^ can result from the stretching vibrations of the C=O group in ketones and amide I (C=O stretching vibration in combination with an NH_2_ deformation vibration), but may also contain the contribution of C=C and C=N vibrations [49]. The range of 1150–1000 cm^−1^ poses challenges in accurate interpretation due to the overlapping absorption of C–O stretching vibrations and C-OH bending vibrations in numerous organic compounds (e.g., ethers, esters, alcohols and amides). The modes are interconnected with additional vibrations and lack clearly defined group frequencies [50]. Moreover, the O–H stretching observed in the mineral components can be associated with the bands within the range of 1170 to 1000 cm^−1^. The band at 950 cm^−1^ corresponds to out-of-plane C-H and/or O-H···O deformation vibrations in carboxyl groups. The peak at ~618 cm^−1^ is related to C=O wagging vibration or N-C=O deformation vibrations in amides and the peaks at 521 cm^−1^ and 465 cm^−1^ are the result of C-C skeleton vibration in aliphatic chains [46].

As already mentioned, the chemical composition of car wash wastewater is complex. The bands visible in the IR spectra contain contributions from surfactants originating from cleaning agents, but also from oils, greases, petrol or diesel residues, etc. The vibration bands of the C-C and C-H groups are relatively high, which confirms the presence of aliphatic chains. These in turn may be due to the presence of both organic pollutants and surfactants. The cavitation process conducted at 3 bar leads to a stronger decomposition of the aliphatic C-C and C-H groups than a pressure of 5 bar, which is indicated by a slight decrease in the vibration bands of these groups in the spectra shown in Figure 3a. In addition, the surfactants decompose at both pressures (reduction in the intensity of the bands within the 1450–1000 cm^−1^ range), but faster at a pressure of 3 bar (the intensity of the bands is reduced after only 30 min). Analysing the IR spectra, it can be concluded that the cavitation process definitely influences the decomposition of organic substances in car wash wastewater. The aliphatic sodium and ammonium salts of large-chain sulfonic acids are often used as surfactants in detergents. Due to the presence of two S=O bonds, the sulfone group is strongly acidic and the degree of acidity is slightly dependent on the substituent type (hydrocarbon chain). Therefore, the decomposition of sulfone groups by the cavitation process reduces the concentration of acid groups in the system, causing the above-mentioned increase in pH and decrease in the specific conductivity of the system.

## 3. Materials and Methods

### 3.1. Materials

The real industrial wastewater came from the wastewater tank of a self-service car wash (Lublin, Lublin Voivodeship, Poland). To ensure accuracy, the experiment was repeated three times for each inlet pressure using a 35 L sample. Table 4 lists the main parameters characterising the wastewater. The data are the averaged values determined during the experiments conducted in triplicate.

### 3.2. Operational Set Up and Experimental Installation

The closed-loop experiment involved the utilization of a pressure-generating pump and a cavitation device, both connected to a circulation tank with a volume of 30 L. The cavitation inductor consisted of a steel plate measuring 64 mm in outer diameter, featuring 9 holes with a diameter of 1 mm each at the centre. For a comprehensive understanding of the laboratory setup, please refer to [51]. This study evaluated the impact of inlet pressure and cavitation duration on surfactant reduction by sampling at 30, 60, 90 and 120 min intervals. Inlet pressures of 2, 3, 4 and 5 bar were tested, with the number of passes through the cavitation zone outlined in Table 5. The cavitation number (c_v_) was determined using the formula c_v_ = (p_2_ − p_v_)/(0.5·ρ·v_0_^2^), where p_2_ represents the pressure behind the nozzle, p_v_ is the vapor pressure of the liquid, ρ is the liquid density and v_0_ is the flow velocity through the orifice. The cavitation number values for each pressure setting can be found in Table 5. It is worth noting that the hydrodynamic cavitation system operates without a cooling system, resulting in a temperature increase during the process.

### 3.3. Physicochemical Measurements of the Wastewater

The analyses of surfactant concentrations, turbidity, phosphates and COD were carried out using medium and standard cuvette tests (Hach Lange, Berlin, Germany), which correspond to a given parameter. The energy consumption was determined using data from the inverter controlling the pump, which recorded the voltage, current and speed so that the actual motor power could be calculated individually for each trial. The measurements were repeated three times for each experimental time and the average values are reported.

Measurements of the surface tension of the wastewater samples before and during the cavitation treatment (0, 30, 60, 90 and 120 min) were conducted at a temperature of 25 °C with a K9 tensiometer (Krüss, Hamburg, Germany) using the ring method. Prior to each measurement, the platinum ring was subjected to cleaning and flame-drying. Then, it was immersed in the system (50 cm^3^) to be measured and then pulled out to measure the surface tension. The surface tension values were calculated by utilizing the maximum force necessary to pull the ring through the interface. The measurements were repeated six times and the average values are reported.

The pH of the wastewater was monitored before and during the cavitation treatment process at different time points (0, 30, 60, 90 and 120 min) using a CX-401 pH meter manufactured by Elmetron in Zabrze, Poland. The EPS-1 electrode was submerged directly into the solution for accurate readings. The experiments were carried out at a temperature of 25 °C and were repeated three times. Calibration of the pH meter was done before each round of measurements. The CX-401 conductivity meter (Elmetron, Poland) was used to measure the specific conductivity of the wastewater before and during the cavitation treatment. The measurements were taken at different time intervals (0, 30, 60, 90 and 120 min) by immersing the EC-60 electrode directly into the wastewater (50 cm^3^). The conductivity meter was calibrated before each series of measurements and the entire process was conducted at a temperature of 25 °C. The measurements were repeated three times to ensure accuracy.

The zeta potential of the wastewater was measured before and during the cavitation treatment at specific time intervals (0, 30, 60, 90 and 120 min) using a NanoZS Zetasizer provided by Malvern Instruments, Malvern, UK. A universal dip cell containing 2 mm palladium electrodes and PCS1115 cuvettes was employed for the analysis. The samples were loaded into a pre-rinsed folded cell for zeta potential measurements. The zeta potential values were calculated based on the electrophoretic mobility data using the Smoluchowski equation. All measurements were performed at a temperature of 25 °C and repeated six times. The average particle size (z-ave) of the wastewater samples was determined before and during the cavitation treatment process. The measurements were conducted using a NanoZS Zetasizer (Malvern Instruments, UK) with a back-scattering detector (173 degrees) and dynamic light scattering (DLS) technique. The samples were analysed in disposable polystyrene cuvettes at a temperature of 25 °C. To ensure statistical significance, all data were collected in automatic mode, repeating each measurement six times to accumulate an adequate number of photons.

The viscosity of the wastewater samples was determined using an Anton Paar viscometer, AMVn (Graz, Austria), before and during the cavitation treatment at specific time intervals (0, 30, 60, 90 and 120 min). The volume of the analysed sample was approximately 50 cm^3^. The precision of the measurements was 0.0001 mPa·s with an uncertainty of 0.3%. The tests were conducted at 25 °C and repeated three times.

FT-IR/ATR analysis was carried out to examine variations in organic material in the wastewater, which could be attributed to cavitation. A Nicolet 6700 spectrometer (Thermo Fisher Scientific, Waltham, MA, USA) and Meridian Diamond ATR accessory (Harrick Scientific Products, Inc., Pleasantville, NY, USA) were used to record the spectra. Prior to FT-IR/ATR analysis, the samples were left and subjected to sublimation (freeze-drying). For a more in-depth explanation of the research methodology, please refer to [52].

### 3.4. Methodology of Conducting Multi-Criteria Decision Support

The optimal HC conditions from twenty tested variants were determined using a multi-criteria decision support procedure (Table 1). The defined criteria included the concentration of cationic, anionic and non-ionic surfactants as well as COD and energy consumption by HC, all of which were minimised. The inlet pressure and the test duration were assumed as variables for the respective variants, which strictly relate to the number of passes of the wastewater through the cavitation zone (Table 5). The analysis was conducted for a five-dimensional criteria space. At the initial stage of the analysis (AS-1), Pareto-optimal solutions that did not dominate were found. Then, the Chebyshev metric was used to generate a first-order (optimal) compromise solution; this formed the second stage of the analysis (AS-2). The reference values were assumed to be the minimum for the specified criteria and formed an ideal vector. Such an approach enabled a conversion of the target values into dimensionless quantities and thus a comparison between the variants. Both min-max analysis and min-max analysis with weights were performed, the first using the same weights (wi) for each criterion (each weight of 0.2, the sum of all weights equalled 1) while the second used various weights reflecting the importance of each criterion. The final stage (AS-3) consisted of selecting the subset of (optimal) compromise solutions [53] using the above-mentioned metric and the same weights for the importance of the criteria, as previously adopted. At this stage, new ideal vectors were created by aggregating the components of an ideal vector with the compromise solution, all of which came from AS-2. These ideal vectors can be used to identify other (sub-optimal) second-order compromise solutions so that other recommended variants can be displayed.

## 4. Conclusions

The results obtained confirm that hydrodynamic cavitation is a promising technique that might be applied for the simultaneous removal of different groups of surfactants from real car wash wastewater. To select the optimal conditions for HC, the multi-criteria decision support was used. The removal of surfactants and COD as well as energy input were chosen as the main criteria of this evaluation. The results of the multi-criteria decision showed that the most beneficial variants involved the inlet pressure of 3 and 5 bar. The highest reduction of non-ionic surfactants was observed at an inlet pressure of 5 bar and reached 35.5% after 120 min of the process. For the removal of anionic surfactants, the most favourable inlet pressure was 3 bar and the removal efficiency was 77.2% after 120 min of the process, whereas the most favourable inlet pressure for cationic surfactant removal was 3 bar with the highest removal of 20% after 120 min. The achieved results demonstrate that HC can be an effective and energy-efficient method for the removal of surfactants from industrial wastewater. Future prospects are to test the suitability of other inlet pressures and to test the suitability of HC for other industrial wastewater, the composition of which varies depending on the production process, weather conditions or place of origin.

## Figures and Tables

**Table 1:** Energy consumption for specific experimental variants.

Time min	Inlet Pressure							
	2 bar		3 bar		4 bar		5 bar	
	Variant Number	Energy Consumption kWh	Variant Number	Energy Consumption kWh	Variant Number	Energy Consumption kWh	Variant Number	Energy Consumption kWh
0	v1	-	v6	-	v11	-	v16	-
30	v2	0.182	v7	0.224	v12	0.279	v17	0.332
60	v3	0.361	v8	0.448	v13	0.555	v18	0.664
90	v4	0.546	v9	0.672	v14	0.833	v19	0.996
120	v5	0.723	v10	0.896	v15	1.11	v20	1.328

**Table 2:** Multi-criteria analysis—arrangement and results.

Stage of analysis	AS-1	AS-2/1	AS-2/2	AS-2/3	AS-2/4
Weights	-	w_1_ = w_2_ = w_3_ = w_4_ = w_5_ = 0.2	w_1_ = 0.2, w_2_ = 0.3, w_3_ = 0.1, w_4_ = 0.15, w_5_ = 0.25	w_1_ = 0.1, w_2_ = 0.1, w_3_ = 0.1, w_4_ = 0.1, w_5_ = 0.6	w_1_ = 0.5, w_2_ = 0.1, w_3_ = 0.1, w_4_ = 0.1, w_5_ = 0.2
Number of variants recommended	v1–v12, v14–v20	v10	v10	v10	v10
Comments	Non-dominated variants	First-order compromise variant min-max	First-order compromise variant min-max with weights		
Stage of analysis		AS-3/1	AS-3/2	AS-3/3	AS-3/4
Weights		w_1_ = w_2_ = w_3_ = w_4_ = w_5_ = 0.2	w_1_ = 0.2, w_2_ = 0.3, w_3_ = 0.1, w_4_ = 0.15, w_5_ = 0.25	w_1_ = 0.1, w_2_ = 0.1, w_3_ = 0.1, w_4_ = 0.1, w_5_ = 0.6	w_1_ = 0.5, w_2_ = 0.1, w_3_ = 0.1, w_4_ = 0.1, w_5_ = 0.2
Number of variants recommended		v17	v17	v17	v17, v9
Comments		Second-order compromise variant min-max	Second-order compromise variant min-max with weights		

**Table 3:** Physicochemical analysis of the wastewater under optimal HC conditions (average values and standard deviations are given).

		pH	Viscosity [Pa·s]	Surface Tension [mN/m]	Specific Conductivity [mS·cm^−1^]	Zeta Potential [mV]	Particle Size [nm]
	
Time		3 bar					
0		7.56 ± 0.10	9.8 × 10^−4^ ± 2.4 × 10^−5^	59.58 ± 2.4	1.14 ± 0.18	−11.70 ± 0.74	1560.0 ± 556
30		8.11 ± 0.05	9.3 × 10^−4^ ± 3.0 × 10^−5^	66.48 ± 1.5	1.12 ± 0.18	−12.15 ± 0.58	1306 ± 464
60		8.34 ± 0.08	9.5 × 10^−4^ ± 2.7 × 10^−5^	69.13 ± 1.7	1.11 ± 0.17	−12.56 ± 0.85	1164 ± 408
90		8.41 ± 0.13	9.8 × 10^−4^ ± 5.8 × 10^−5^	71.49 ± 0.9	1.08 ± 0.16	−12.89 ± 0.98	1008 ± 328
120		8.45 ± 0.16	9.9 × 10^−4^ ± 2.6 × 10^−5^	72.45 ± 0.5	1.06 ± 0.016	−13.11 ± 1.08	937 ± 128
Time		5 bar					
0		7.54 ± 0.09	9.4 × 10^−4^ ± 2.3 × 10^−5^	65.93 ± 2.2	1.06 ± 0.3	−12.03 ± 0.13	1065 ± 205
30		8.26 ± 0.07	9.9 × 10^−4^ ± 6.4 × 10^−5^	70.44 ± 0.2	1.05 ± 0.2	−12.13 ± 0.52	917 ± 136
60		8.46 ± 0.07	9.3 × 10^−4^ ± 5.4 × 10^−5^	71.6 ± 0.2	1.03 ± 0.1	−12.28 ± 0.74	876 ± 125
90		8.65 ± 0.08	9.3 × 10^−4^ ± 3.9 × 10^−5^	71.81 ± 0.5	1.00 ± 0.5	−12.63 ± 1.08	813 ± 78
120		8.68 ± 0.05	1.0 × 10^−3^ ± 3.4 × 10^−5^	72.2 ± 0.1	0.97 ± 0.7	−12.8 ± 0.45	819 ± 59

**Table 4:** Parameters characterizing wastewater for the analysed inlet pressure (including standard deviation and average values).

Parameter	Inlet Pressure [bar]			
	2	3	4	5
NS concentration [mg L^−1^]	164 ± 3.3	153 ± 3.1	140 ± 2.8	204 ± 4.1
AS concentration [mg L^−1^]	39.5 ± 2.0	50.5 ± 2.5	36.9 ± 1.8	40.6 ± 2.0
CS concentration [mg L^−1^]	0.87 ± 0.043	0.58 ± 0.058	1.18 ± 0.094	0.5 ± 0.035
COD [mg L^−1^]	251.1 ± 5.0	159 ± 3.2	165 ± 6.3	249 ± 7.5
pH -	7.51 ± 0.11	7.77 ± 0.13	7.76 ± 0.12	7.49 ± 0.11
Turbidity [NTUs]	56.3 ± 0.1	43.3 ± 0.2	35.7 ± 0.2	53.7 ± 0.1
Phosphates [mg L^−1^]	0.203 ± 0.011	0.189 ± 0.01	0.178 ± 0.013	0.216 ± 0.01

**Table 5:** The cavitation number values and number of passes through the cavitation zone for the analysed inlet pressures.

Parameter	Inlet Pressure [bar]			
	2	3	4	5
p_v_ [kPa]	2.337	2.642	2.642	2.485
p_2_ [kPa]	96.67	96.85	96.89	96.94
v_0_ [ms^−1^]	32.49	36.77	46.05	51.21
c_v_ -	0.18	0.12	0.09	0.07
Time [min]	Number of passes through cavitation zone			
30	13.8	16.9	19.5	21.7
60	27.6	33.7	39.1	43.4
90	41.3	50.6	58.6	65.2
120	55.1	67.5	78.1	86.9

## Data Availability

The raw/processed data required to reproduce these findings cannot be shared at this time due to technical or time limitations.
